# Development of a low-dose fipronil deer feed: evaluation of efficacy against two medically important tick species parasitizing white-tailed deer (*Odocoileus virginianus*) under pen conditions

**DOI:** 10.1186/s13071-023-05689-1

**Published:** 2023-03-09

**Authors:** David M. Poché, Donald Wagner, Kylie Green, Zachary Smith, Noah Hawthorne, Batchimeg Tseveenjav, Richard M. Poché

**Affiliations:** 1 Genesis Laboratories, Inc., Wellington, CO USA; 2 Pennsylvania State University, University Park, PA USA

**Keywords:** Blacklegged ticks, *Ixodes scapularis*, Lone star ticks, *Amblyomma americanum*, White-tailed deer, *Odocoileus virginianus*, Fipronil deer feed, Acaricides, Systemic insecticides, Vector control

## Abstract

**Background:**

*Odocoileus virginianus* (the white-tailed deer) is a key reproductive host for medically important tick species, including *Ixodes scapularis* and *Amblyomma americanum*. Orally administering a systemic acaricide to white-tailed deer has the potential to reduce tick reproduction, abundance and pathogen-infected tick bites. Prior studies have demonstrated considerable efficacy of a low-dose fipronil mouse bait in controlling larval *I. scapularis* parasitizing the pathogen reservoir, *Peromyscus leucopus*. No prior studies have evaluated the efficacy of a fipronil product in controlling ticks parasitizing white-tailed deer.

**Methods:**

A pen study was conducted to evaluate the efficacy of a fipronil deer feed in controlling *I. scapularis* and *A. americanum* adult ticks. Individually housed deer (*n* = 24) were exposed to deer feed containing 0.0025% fipronil (fipronil deer feed) for 48 h and 120 h, and a control group of deer were exposed to an untreated placebo. On post-exposure day 7 and day 21, all deer were parasitized with 20 mating pairs of feeding capsule-enclosed *I. scapularis* and *A. americanum*. Post-attachment, engorgement and mortality of ticks were recorded. The concentrations of fipronil in plasma, feces and tissues from euthanized deer were estimated using liquid chromatography-mass spectrometry.

**Results:**

The fipronil deer feed efficaciously controlled ticks parasitizing pen-reared white-tailed deer. Efficacy in reducing survivorship of blood-feeding female *I. scapularis* exceeded 90% in all instances except for when ticks parasitized 48-h treated deer at day 21 post-exposure (47.2%). Efficacy in reducing survivorship of *A. americanum* females exceeded 80% in all instances. In the 120-h exposure group there was 100% tick mortality at day 7 post-exposure for both tick species. A significant correlation was observed between reductions in tick survivorship and concentrations of fipronil sulfone in plasma. The results of tissue analysis suggest that a withdrawal period may be needed to allow for fipronil degradation prior to hunting season.

**Conclusions:**

The results provide proof-of-concept for the use of a fipronil-based oral acaricide in controlling two medically important tick species infesting a key reproductive host. A field trial is necessary to confirm the efficacy and toxicology of the product in wild deer populations. Fipronil deer feed may provide a means of controlling multiple tick species parasitizing wild ruminants to be integrated into tick management programs.

**Graphical Abstract:**

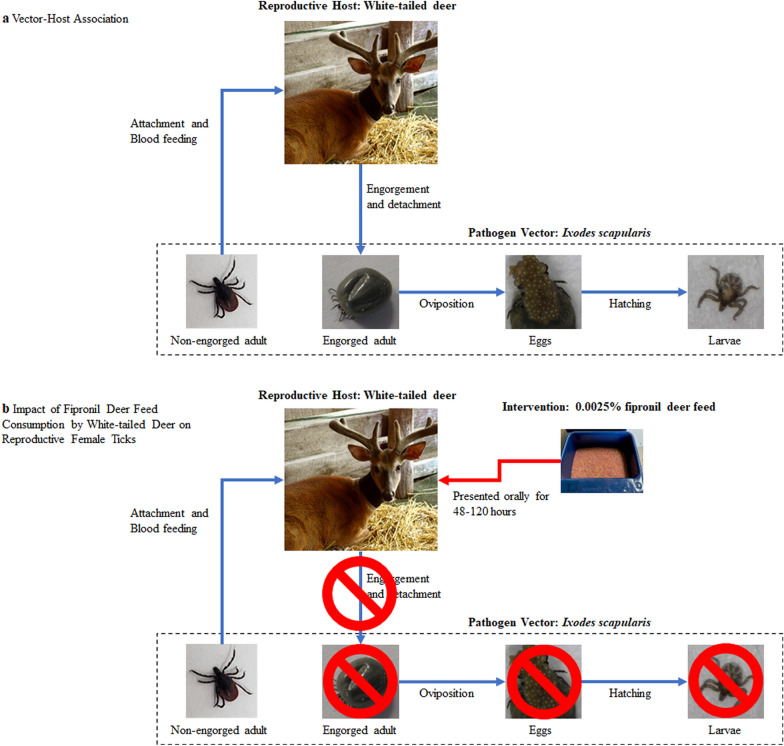

**Supplementary Information:**

The online version contains supplementary material available at 10.1186/s13071-023-05689-1.

## Background

On a global scale, ticks are recognized as one of the main arthropod pathogen vectors of disease agents of humans and animals, and thus are of considerable medical importance [1]. Ticks and wildlife species encompass vector-host relationships of increasing medical and veterinary concern, with many notable tick-borne diseases, such as anaplasmosis, babesiosis, ehrlichiosis and Lyme disease, attracting substantial medical attention [2]. Vector control is regarded as one of the more promising means for reducing human tick bites and preventing pathogen transmission. However, conventional methods, such as area-wide broadcast applications, present management concerns, including logistical and economic hurdles, the indiscriminate targeting of non-target organisms, such as pollinators, and the accelerated development of insecticidal resistance [3–5]. Thus, additional, more discriminate methods of control should be explored to supplement conventional practices.

White-tailed deer serve as a potential blood-meal host for several medically important tick species, including *Ixodes scapularis* (blacklegged tick), *Amblyomma americanum* (lone star tick) [6], *Haemaphysalis longicornis* (Asian longhorned tick) [7] and *Rhipicephalus* *microplus* (cattle fever tick) [8]. An exponential increase in white-tailed deer populations and geographical distribution has been linked to an increase in *I. scapularis* abundance and distribution [9] and the subsequent rise in the incidence of Lyme disease [10, 11]. This in turn can be attributed to white-tailed deer representing the primary breeding sites of *I. scapularis* [12], with approximately 90% of adult *I. scapularis* being estimated to feed on deer [9]. Thus, white-tailed deer represent a key reproductive host for this tick species. The increase in deer populations has also been linked with an increase in *A. americanum* populations [13], a medically important tick species which parasitizes white-tailed deer at multiple life stages (adults, nymphs, larvae) [14] and is heavily reliant on this host for reproduction and development. Attempts have been made to control parasitizing ticks by targeting white-tailed deer with topical acaricides, using the federally approved permethrin-based product, the 4-Poster Tick Control Deer Feeder [15]. The device is filled with untreated corn and when deer access the corn permethrin is topically applied to them by paint rollers. A number of issues have limited the use of this technology, including the labor and maintenance required to service the device (refilling corn, applying permethrin to rollers, fixing broken rollers, etc.). Although studies have suggested that this technology is promising in terms of tick control, there has been a lack of success in impacting overall instances of Lyme disease [16]. A more direct, practical and less cumbersome approach would be to present the deer with a feed containing an oral acaricide.

Oral acaricides represent a more direct means of delivering acaricides to deer and they act systemically, with small quantities of the acaricide being consumed by ticks during blood-feeding [17]. A previous effort was made to control *I. scapularis* by targeting deer with ivermectin-treated corn in an island scenario in Maine [18]. While the results demonstrated the ability of systemic ivermectin to control blood-feeding ticks when it was detectable in plasma at ≥ 15 parts per billion (ppb), no impact was observed on the presence of host-seeking ticks in the treatment area, relative to control. Thus, it may be advantageous to investigate other alternative acaricidal compounds.

The phenylpyrozol, fipronil, interferes with the central nervous system in arthropods through the blockage of GABA-gated and glutamate-gated chloride channels [19]. Authors of previous research evaluating candidate acaricidal compounds (including fipronil and ivermectin) concluded that fipronil demonstrated superior effectiveness and longevity in controlling ticks and fleas [17–20], phlebotomine sand flies [21] and mosquitoes [22], suggesting that it might be a useful means of targeting white-tailed deer to control blood-feeding ticks. The majority of the systemic work with fipronil in the USA has focused on controlling *Oropsylla* spp. fleas feeding on black-tailed prairie dogs [23–25] and controlling *I. scapularis* feeding on *Peromyscus leucopus* (white-footed mouse) [26, 27], a host species which represents the principal pathogen reservoir for *Borrelia burgdorferi* in Northeast and Midwest USA. The latter research resulted in the development of a fipronil-based bait (0.005% fipronil) that was capable of controlling up to 100% blood-feeding *I. scapularis* larvae parasitizing *P. leucopus* for up to 15 days post-exposure under laboratory conditions [26], and up to 35 days post-exposure under simulated field conditions [27]. The results of the latter experiment led the authors to conclude that 100% efficacy was attainable if mice had fipronil sulfone present in plasma at concentrations of ≥ 8.8 ppb.

While the above product may prove useful in targeting a principal pathogen reservoir of Lyme disease spirochetes in Northeast and Midwest USA, adult *I. scapularis* do not feed on rodents and thus would not be targeted with this approach. Considering the fact that 90% of *I. scapularis* ticks have been estimated to parasitize and feed upon white-tailed deer [9], directly targeting this host with an acaricide could significantly reduce the reproductive success of this tick species. Additionally, other tick species, such as *A. americanum*, which are less reliant on rodent species, but heavily reliant on white-tailed deer for development and reproduction, would be targeted by this approach. Thus, targeting white-tailed deer with a fipronil-based oral acaricide has the potential to have an impact on multiple medically important tick species, including those not reached by rodent-targeted approaches.

The absence of available scientific literature relating to the control of ticks in white-tailed deer with fipronil strongly suggests that no oral fipronil products have been evaluated for the control of tick vectors parasitizing white-tailed deer in the USA. However, extensive work has been conducted internationally, with evaluations on the use of fipronil in controlling phlebotomine sand flies and *Anopheles* spp. mosquitoes blood-feeding on cattle [22, 28–30]. The reported efficacy of the fipronil products used in these studies indicates that fipronil formulations can be successfully delivered to ruminant species to control a variety of arthropod vectors. Therefore, in the present study, an oral acaricide feed (0.0025% fipronil; referred to hereafter as fipronil deer feed) was developed for delivery to white-tailed deer to control parasitizing ticks. As a precursor to any potential field testing, we conducted the study under pen conditions to determine the palatability of the feed, its efficacy against parasitizing ticks and fipronil residue levels in tissues. These data will provide better insights into effective execution of potential management practices in the future.

## Methods

**Fig. 1:**
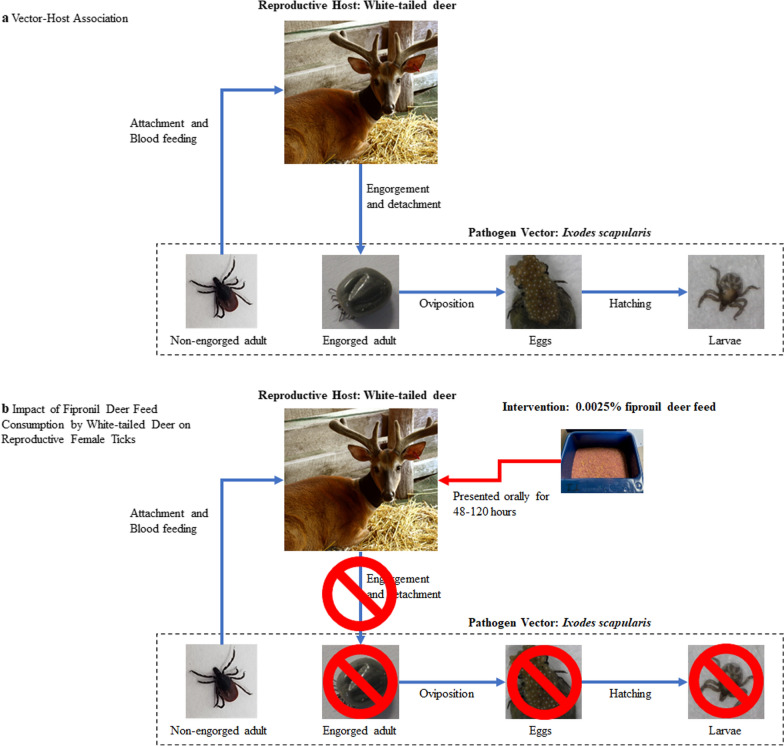
Vector-host association (**a**) and impact of fipronil deer feed consumption by white-tailed deer on reproductive female ticks (**b**). **a** Adult females attach to white-tailed deer and blood feed for approximately 6–11 days. Fully engorged females drop off of the host and begin the reproductive process. Females then oviposit and produce thousands of eggs. **b** Adult female ticks blood-feeding on white-tailed deer expire and are prevented from feeding to engorgement and detaching, subsequently preventing them from successfully ovipositing and reducing the reproductive rate

The primary objective of the study described herein was to evaluate the efficacy of a fipronil deer feed against *I. scapularis* and *A. americanum* ticks parasitizing white-tailed deer under pen conditions. The vector-host association and treatment concept are presented in Fig. 1. *Ixodes scapularis* was selected because it is a vector of seven human pathogens, with the most notable being those causing Lyme disease [31, 32]. Lyme disease is the most common vector-borne disease in the USA, occurring most frequently in the Northeast and Midwest of the USA, and is estimated to account for approximately 500,000 human cases per year [32–34]. *Amblyomma americanum* was selected because it is suspected to vector five or more disease agents transmissible to humans [35], and is also linked with southern tick-associated rash illness (STARI) [36] and red meat allergy [37, 38].

### Study site

The study was conducted during 2021 and 2022. All research involving white-tailed deer over the course of this project was performed at the Pennsylvania State University (PSU) Deer Research Center (State College, PA, USA), a facility that maintains a herd of approximately 75–100 captive white-tailed deer. The facility contains nine large outdoor paddocks for group housing and breeding, and a centrally located handling barn with 24 pens for individual animal housing.

All activities involving animals during this study were performed in accordance with the Animal Welfare Act, the Office of Laboratory Animal Welfare and Pennsylvania State University Institutional Animal Care and Use Committee (IACUC) policies (PSU Protocol No. PROTO202101784, Approval Date: February 15, 2021).

### Fipronil deer feed

Fipronil deer feed (FDF) is a granular formulation developed as part of US Centers of Disease Control and Prevention (CDC) Contract No. 75D30120C09834. As part of the initial acaricide development process, a semi-field screening study was conducted to determine the most palatable formulations, followed by a dose range-finding study involving adult *I. scapularis* to determine the optimal fipronil concentration. This study resulted in the development of FDF, a granular sugar beet formulation that was considerably palatable to white-tailed deer with a nominal fipronil concentration of 0.0025%, which was determined to control 100% *I. scapularis* parasitizing white-tailed deer at 24 h post-exposure when they were presented with FDF for 48 h (Poché et al., unpublished data).

To produce FDF, the raw ingredients were mixed in an industry-standard electronic mixer (Marion Processing Solutions, Marian, IA, USA) capable of holding approximately 225 kg of material. The formulation contained a nominal fipronil concentration of 0.0025% (25 ppm), which was confirmed by the Colorado State University Environmental Medicine Analytical Laboratory (CSU) (Fort Collins, CO, USA) using a validated high-performance liquid chromatography (HPLC) method with a limit of quantification (LOQ) of 5 ppm. The concentration of fipronil in the FDF in two produced batches was 28.2 ± 0.71 and 26.2 ± 0.69 ppm, respectively.

### Experimental design

#### Pre-fipronil deer feed exposure (acclimation)

Both male and female adult and yearling test deer were utilized. At the initiation of acclimation, test deer were transferred from group paddocks into the handling barn where they were maintained individually in approximately 7.5 (length) × 3-m (width) pens. The roofs of the pens were partially opened, allowing for sunlight, but also had canopies to shield the test deer and FDF from inclement weather. Weather data were collected from the Patton Township weather station in State College, PA (Station ID: KPASTATE15) over the course of the project. The walls of the pens were high enough to prevent deer from escaping (approx. 3 m). Deer were acclimated to test conditions for 3 days prior to FDF exposure, and the general health of all deer was monitored daily. During this time, deer were presented with commercial deer diet (PSU Breeder 18% Deer Diet; Cargil Animal Nutrition, Minneapolis, MN, USA) ad libitum and each day were presented with approximately 500 g of untreated feed containing all of the inactive ingredients in the FDF (placebo). All deer were examined by a veterinarian prior to FDF exposure.

#### White-tailed deer test group and subgroup assignment

Deer were assigned to groups using a random sequence generator, and groups were differentiated based on: (i) test group identity (treatment group [T], control [C]); and (ii) the length of the exposure (48 h [T48], 120 h [T120]) (Additional file 1: Table S1). While explicit guidelines for white-tailed deer are not available, federal guidelines recommend a sample size of 6–10 subjects per test group when evaluating pesticides against pests of humans and pets, such as fleas and ticks [39]. The size of the captive herd and the number of deer that its managers could afford to donate to this project limited the sample size that we were able to utilize, and we were unable to have an equal number of males (*n* = 15) and females (*n* = 9). It was determined that each test group could comprise eight animals (*n* = 24). A total of 16 deer were offered FDF, with eight deer being exposed to FDF for 48 h and eight deer being exposed to FDF for 120 h. An additional eight deer served as an untreated control group, with 50% of animals exposed to placebo for 48 h and 50% exposed to placebo for 120 h. Deer continued to be housed in the individual pens during the exposure period. Prior to tick attachment, deer within each test group were additionally assigned to subgroups, with 50% of deer to be parasitized with ticks at day 7 post-exposure to FDF, and 50% to be parasitized at day 21 post-exposure to FDF (4 animals/subgroup).

#### FDF exposure

At the initiation of the deer feed exposure period, deer were presented exclusively with FDF in an elevated livestock feeder (Additional file 2: Figure S1). A maximum of 1 kg was presented to deer every 24 h, and deer were provided commercial deer diet immediately upon consuming all FDF. Each morning of exposure (08:00 a.m.) the FDF was removed temporarily and weighed to the nearest 0.1 g, after which fresh FDF was immediately presented to the deer. At the conclusion of the exposure period, all FDF was weighed and permanently removed. The above procedures were also followed for the deer in the control group, but they were presented with an untreated placebo (containing all ingredients of FDF minus fipronil) rather than FDF.

#### Post-deer feed exposure deer handling

At the conclusion of exposure, all FDF or placebo was removed, and deer were released into group paddocks until tick attachment. Deer were fed on a commercial diet exclusively ad libitum for the remainder of the study. During the post-exposure period, but prior to tick attachment, deer remained in the group paddocks and their general health was observed daily.

#### Tick attachment

Ticks were acquired from the Oklahoma State Tick Rearing Facility (OSU) (Stillwater, OK, USA). Equal numbers of each sex and species (*I. scapularis* and *A. americanum*) were obtained. For each lot of *I. scapularis* and *A. americanum* and prior to shipment to the study site, OSU screened a subsample of ticks (*n* = 10) for pathogens using standardized PCR assays. *Ixodes scapularis* were screened for *B. burgdorferi* and *Anaplasma phagocytophilum. Amblyomma americanum* were screened for the presence of *Ehrlichia chaffeensis*, *Francisella tularensis* and *Rickettsia rickettsii*. All PCR-screened ticks were negative for the above pathogens. Once ticks arrived at the study site, they were housed in an industry-standard desiccator with the relative humidity maintained at > 90% until enclosed in a feeding capsule for attachment to deer.

The feeding capsules utilized in this study were specifically designed for holding blood-feeding *I. scapularis* and *A. americanum*. Feeding capsules allow for the containment and localization of ticks and aid in facilitating blood-feeding [40]. The traditional stockinet sleeve method for feeding ticks on cattle [41–43] was determined to be inadequate for white-tailed deer. We instead developed a feeding capsule for deer application, which was in part based upon feeding capsules for ticks (referred to hereafter as tick feeding capsules) previously designed for tick-feeding on rabbits and sheep [44]. To make each capsule, sheets of ethylene–vinyl acetate foam were cut into three square pieces. Each square had a different outside area, allowing for flexibility (base, approx. 12 × 12 cm; middle, approx. 9 × 9 cm; top, approx. 7 × 7 cm), and had a combined depth of approximately 18 mm. The center of each square was cut away, creating an opening. The inner surface areas of the base and middle piece openings were each approximately 7 × 7 cm; the top piece had a smaller opening (approx. 1.5 × 1.5 cm) through which the ticks were to be inserted, which decreased the probability that ticks would escape through the top of the capsule (Additional file 3: Figure S2).

Deer were anesthetized using an intramuscular injection of telazol and xylazine at dosages of approximately 3 mg/kg and approximately 2.5 mg/kg, respectively. Once fully anesthetized, deer were weighed to the nearest 0.1 kg using a certified balance. Prior to blood collection and capsule attachment, large patches of fur on the neck were trimmed using electric horse clippers (Wahl®; Wahl Clipper Corp., Sterling, IL, USA). Prior to capsule attachment, 10 ml of blood was collected from the jugular vein of each deer using a 20-gauge needle. The blood from each individual deer was immediately placed into a vacutainer containing EDTA and was centrifuged for 10 min at 7000 revolutions/min. The plasma was transferred to 1.5-ml centrifuge tubes, which were then stored at − 20 °C until analysis.

**Fig. 2:**
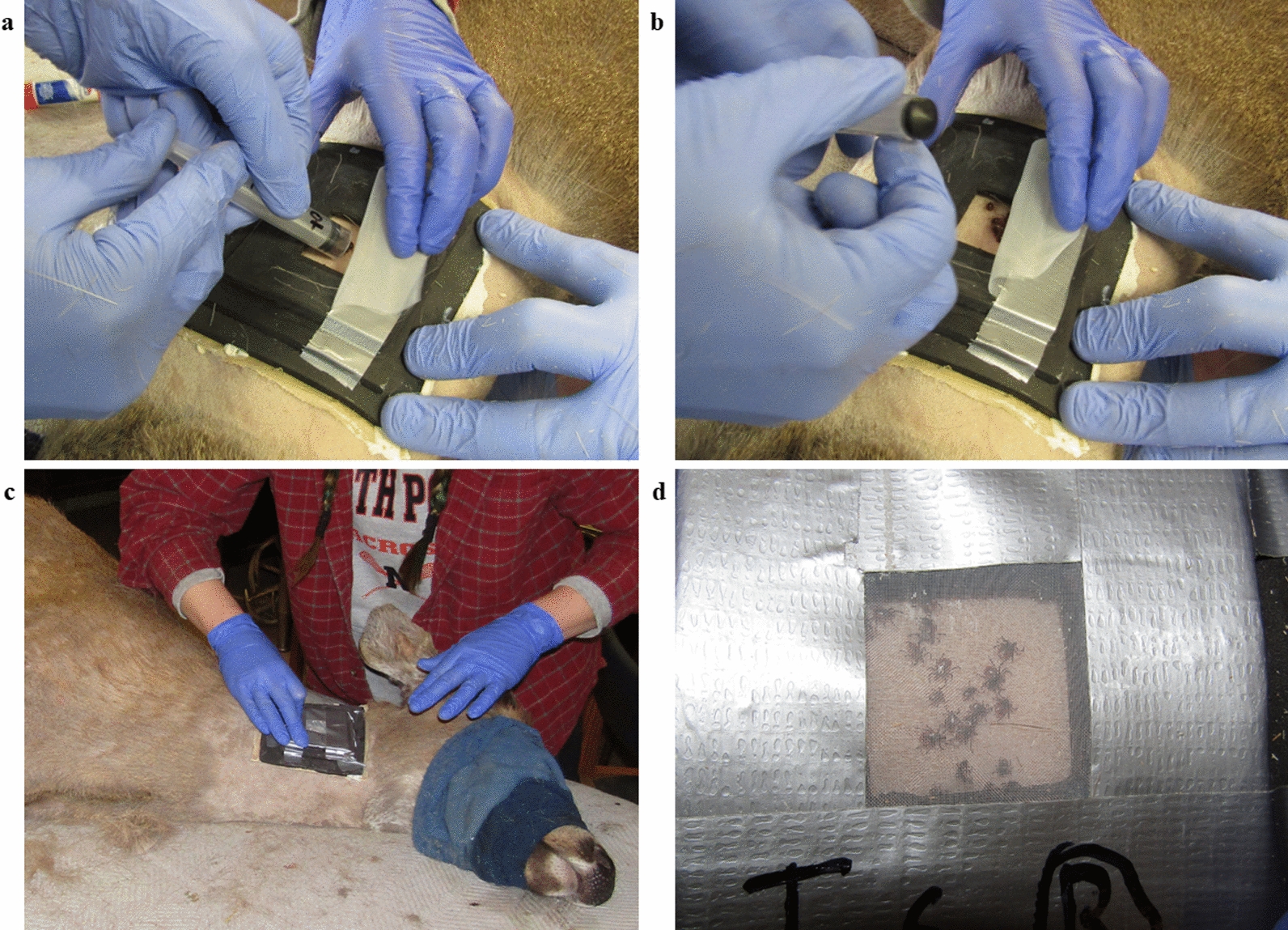
Tick capsule attachment and tick attachment. **a** Female ticks being plunged into capsule, **b** plunger being removed prior to mesh lid being secured, **c** completed, secured capsule being checked to ensure all corners are adhered to the neck, **d** closeup of completed capsule containing 20 *Ixodes scapularis* mating pairs

Two identical tick feeding capsules were attached to opposing sides of the neck of each deer using a liberal amount of fabric glue (Tear Mender, St. Louis, MO, USA). Each capsule was held firmly in place for > 3 min to allow it to adhere to the skin and fur. For each deer, 20 *I. scapularis* mating pairs were placed within one capsule, and 20 *A. americanum* mating pairs were placed within the second capsule. Prior to tick attachment, 20 ticks (all same species and sex) were placed into a modified 5-ml syringe. Ticks were chilled in ice for approximately 5–10 min to slow movement. The 20 mating pairs were then carefully plunged into the capsules and a fine mesh lid was applied and reinforced with duct tape. Representative photos and video of the tick attachment process are presented in Fig. 2 and Additional file 4: Video S1, respectively. The capsules were further secured to deer by wrapping the neck with a veterinary bandage (3 M Company, St. Paul, MN, USA).

After completion of capsule and tick attachment, deer were given tolazine via intramuscular injection at a dose of 4 mg/kg to reverse the effects of the anesthetic. Deer were then housed in individual pens, observed closely until they were mobile and moving normally and monitored routinely for the remainder of the day.

#### Post-attachment

The post-attachment period spanned the initial 8 days following tick attachment (day 0 to day 8). During this time, deer were housed individually in pens (Additional file 5: Figure S3) and checked daily to ensure adequate health and wellbeing and to ensure that the capsules remained firmly attached. At day 6 and day 8 post-attachment, the deer were anesthetized in the previously described manner, and the capsules were opened to monitor the condition of the ticks. These time points were selected because *I. scapularis* was the primary species of concern and reportedly takes approximately 6–11 days to reach engorgement and detach [45]. A minimum of 48 h was required between sedations for each deer because of PSU IACUC policy forbidding sedation of animals on consecutive days.

Ticks were easily visible by the naked eye. The inside of each capsule was carefully scanned for attached and detached ticks. The numbers of total ticks recovered and their attachment status (attached, detached), feeding status (flat, partially engorged, fully engorged) and condition (alive, dead) were recorded. Any dead ticks (attached, detached) were removed from deer. At the conclusion of tick observations on day 8 post-attachment, capsules were completely removed, and any live or dead ticks were manually removed from the deer.

Fully engorged, live detached female ticks were collected, weighed to the nearest 0.0001 g using an analytical balance (Mettler-Toledo, LLC, Columbus, OH, USA), and maintained individually in vials. Engorged females were maintained in a desiccator (> 90% relative humidity) and were allowed approximately 14–28 days to complete oviposition [45]. After oviposition was completed, females were removed, and egg masses weighed to the nearest 0.0001 g. Egg masses were monitored for the emergence of larvae, with eggs embryonating within approximately 35–50 days [45]. Egg masses were monitored for approximately 2–3 weeks to estimate the proportion of hatched eggs.

#### Deer tissue collection

At the conclusion of the tick observations on day 8 post-attachment, fresh fecal samples were collected from each test deer pen. Additionally, internal tissues were collected from each deer in each treatment group. The deer were first sedated by injection of 1–2 mg/kg xylazine hydrochloride (100 mg/ml) into the large muscle bellies of the rump/rear limbs. While sedated, deer were euthanized by intravenous injection, administered via the jugular vein, of 86 mg/kg Euthasol (pentobarbital sodium, 390 mg/ml), resulting in pentobarbital sodium overdose. Death was confirmed by a combination of the following: (i) lack of heartbeat based on auscultation with a stethoscope; (ii) lack of respiration based on visual inspection of the thorax; (iii) lack of corneal reflex; and (iv) lack of response to firm toe pinch. All euthanasia was performed by the attending veterinarian exclusively.

Various tissues were collected from euthanized deer. The objective was to collect tissues similar to what would be collected by hunters when field dressing a killed deer. Thus, we focused on specific meat cuts, meat by-products and fatty tissues. Approximately 50 g of each tissue was surgically removed using disposable scalpels. Scalpels and surgical gloves were replaced between each individual tissue collection to minimize the risk of contamination. Each tissue was transferred to an individual biological specimen bag (Keefitt®), which was immediately stored at − 20 °C until analysis. In addition to collecting tissues from 16 deer in the treatment group, we collected tissues from two deer in the control group to establish a baseline and for analytical method development.

Tissues, plasma and feces were delivered to CSU for method development and analyses, and analyzed for the presence of fipronil and fipronil metabolites using validated methods of liquid chromatography/mass spectrometry (LC/MS). A list of tissue classifications, the maximum residue limits (MRL) listed by the US Environmental Protection Agency (EPA) for fipronil in cattle and the explicit tissue identifications are presented in Additional file 6: Table S2.

Critical study dates for each test deer (acclimation, exposure, post-attachment, capsule checks, tissue collection) are presented in Additional file 7: Table S3.

### Data analyses

#### FDF consumption and deer body weights

The amount of FDF consumed by each deer was calculated daily and recorded to the nearest 0.1 g. The total fipronil consumed by each deer (mg) and body weights (kg) recorded prior to tick attachment were used to estimate the amount of fipronil (in mg) consumed per kilogram deer. Differences in daily FDF/placebo consumption and deer body weights between groups were compared using an analysis of variance. Differences in total fipronil consumed per deer (mg/kg) were compared between T48 and T120 using a Student’s t-test.

#### Tick observations and recovery

Adult ticks observed and counted at day 6 and day 8 post-exposure were defined by attachment status (attached, detached) and feeding status (non-engorged, partially engorged, fully engorged), with ‘attached’ = adults which remain imbedded in the skin of deer; ‘detached’ = adults which are not imbedded in the skin of deer; ‘flat’ = non-engorged adults, showing no discernable blood meal; ‘partially engorged’ = adults with partial blood meal discernable, but not fully fed; and ‘fully engorged’ = completely bloated and darkly colored adults. Ticks were further defined by condition (dead, alive), which was determined by carefully observing and manipulating attached and detached ticks with fine-tipped forceps to elicit movement, with ‘alive’ = movement of legs, palps or mouthparts; and ‘dead’ = no movement after approximately 45 s of manipulation. The attachment status, feeding status and condition of female ticks were compared between treatment and control groups. The proportion of ticks recovered within each test group was also investigated. Differences in the proportion of ticks attached and detached for each species and differences in feeding status and condition of each species within each test group were compared using a Pearson’s χ^2^ test for independence.

The weights of engorged females and approximate number of eggs and hatched larvae were compared between the treatment and control groups. To estimate the approximate number of eggs in each egg mass, an assumption was made that 1 g of ixodid eggs would contain approximately 20,000 individual eggs [46]. The number of hatched larvae was estimated by multiplying the approximate proportion of hatched eggs by the approximate number of eggs [46]. Differences in the weights of engorged females detaching from FDF-treated deer, relative to deer in the control group, and the subsequent numbers of eggs and larvae produced per female were estimated using a Student’s t-test.

#### Mortality estimates

Mortality/efficacy in controlling *I. scapularis* and *A. americanum* was evaluated post-attachment. We used two metrics to evaluate efficacy: (i) the average number of live engorged females successfully detaching by day 8 post-attachment; and (ii) the average survivorship of females (attached and detached) at the conclusion of day 8 post-attachment.

Efficacy of FDF in controlling blood-feeding *I. scapularis* and *A. americanum*, relative to the untreated control groups, was estimated using Abbott’s formula [47]:

$$\mathrm{Efficacy}\, (\%)=100*\left(\frac{C-T}{C}\right)$$

where *T *= *n* treatment group, and *C *= *n* control group.

#### Fipronil concentration in deer samples

The concentrations of fipronil and fipronil metabolites in plasma (*Cp*) (LOQ = 0.04 ppb) and feces (*Cf*) (LOQ = 0.1 ppb) were estimated for each individual deer (*n* = 24). Linear regression (*P* < 0.05) was used to detect a correlation between *Cp* or *Cf* (dependent) and the mg fipronil/kg body weight consumed by white-tailed deer (independent). Linear regression was also used to detect a potential correlation between *Cp* and survivorship of female *I. scapularis* and *A. americanum* ticks.

The concentrations of fipronil and fipronil metabolites within various tissues (*Ct*) were estimated for 16 FDF-treated deer and two control deer (LOQ = 0.04 ppb). Differences in *Ct* values among all tissue classifications (fat, meat, meat by-products, liver) were estimated using a Kruskal–Wallis H-test followed by a Wilcoxon signed-rank test within each pair. Differences in *Ct* values between the T48 and T120 exposure groups estimated for each tissue classification and differences in *Ct* values of each tissue classification within each test subgroup were estimated using a Wilcoxon signed-rank test. The *Ct* was compared with the MRL established by the US EPA for ruminant cattle [47] (meat/muscle = 40 ppb; liver = 100 ppb; meat by-products = 40 ppb; fat = 400 ppb) which are utilized by the US Food and Drug Administration (FDA) when evaluating potential products. The *Ct* values recorded at each time point post-exposure (day 15, day 29) were used to develop exponential equations to approximate the rate of fipronil degradation for each tissue classification as a function of the number of days post-exposure. The equation was formulated as follows, and is functionally similar to equations previously utilized by Poché et al. [29] to represent fipronil degradation in bovid plasma and feces:

$${\text{Fipronil degradation}} = \Theta^{1} *EXP(\Theta .^{2} x)$$

where* Ɵ*^*1*^ = Theta-1 estimate, *Ɵ*^*2*^ = Theta-2 estimate, *EXP* = exponential, *x* = days post-exposure.

All analyses were performed using the current versions of JMP statistical software (version 15) (SAS Institute, Cary, NC, USA) and Microsoft Excel. Differences were considered significant if *P* < 0.05.

## Results

### FDF consumption and white-tailed deer body weights

A total of 24 deer were utilized in this study, and all deer appeared to be healthy throughout the experiment. Individual deer body weights, total FDF consumption and fipronil consumed are presented in Table 1. For the 48-h exposure group, FDF consumption (g), body weight (kg) and fipronil consumption (mg/kg) ranged from 805.5 to 2000 g, from 50.4 to 93.9 kg and from 0.24 to 0.99 mg/kg, respectively. For the 120-h exposure group, FDF consumption (g), body weight (kg) and fipronil consumption (mg/kg) ranged from 1474.1 to 5000.0 g, from 50.9 to 104 kg and from 0.44 to 1.47 mg/kg, respectively.

**Table 1:** Body weights and feed/fipronil consumption recorded for individual white-tailed deer

Test group	Sex	Weight (kg)	Total feed presented (g)	Total feed consumed (g)	Total fipronil consumed (mg)	Amount fipronil per body weight (mg/kg)
T48 (2-day FDF exposure)	Female	83.8	2000.0	2000.0	50.0	0.60
	Male	86.4	2000.0	2000.0	50.0	0.58
	Female	72.2	1000.0	915.0	22.9	0.32
	Male	93.9	1000.0	1000.0	25.0	0.27
	Male	77.9	1000.0	845.2	21.1	0.27
	Female	82.4	1000.0	805.5	20.1	0.24
	Male	50.4	2000.0	2000.0	50.0	0.99
	Female	72.1	2000.0	976.7	24.4	0.34
T120 (5-day FDF exposure)	Male	104.0	5000.0	5000.0	125.0	1.20
	Female	81.8	5000.0	4051.6	101.3	1.24
	Female	56.8	2500.0	2500.0	62.5	1.10
	Female	68.4	2500.0	2479.0	62.0	0.91
	Female	66.3	2500.0	2464.4	61.6	0.93
	Female	62.7	2500.0	2358.8	59.0	0.94
	Female	50.9	5000.0	2984.3	74.6	1.47
	Female	84.1	5000.0	1474.1	36.9	0.44
Control (2-day placebo feed exposure)	Male	99.9	2000.0	1986.5	NA	NA
	Female	78.8	2000.0	1678.1	NA	NA
	Male	74.2	1000.0	1000.0	NA	NA
	Female	52.8	1000.0	1000.0	NA	NA
Control (5-day placebo feed exposure)	Female	69.3	2500.0	2097.9	NA	NA
	Male	83.5	2500.0	2500.0	NA	NA
	Female	91.3	5000.0	3000.3	NA	NA
	Male	79.2	5000.0	4316.1	NA	NA

*FDF* Fipronil deer feed, *NA* not applicable,* T48* treatment group exposed to FDF for 48 h (2 days),* T120* treatment group exposed to FDF for 120 h (5 days)

No significant differences were found when comparing daily FDF and placebo consumption at 48-h exposure and 120-h exposure. No significant differences were detected when comparing individual deer body weights among test groups. As expected, the amount of fipronil consumed (mg/kg) by each deer was significantly higher in T120 (5-day exposure) than in T48 (2-day exposure) (*t*_(13.636)_ = 4.082, *P* = 0.0012).

### Tick observations

#### Tick recovery and attachment status

The system of containing and recovering adult ticks infesting deer was relatively efficient. Tick recovery and attachment status data are explicitly represented in Table 2 and were used to calculate all sums and percentages presented in this section. In total, white-tailed deer were infested with 840 *I. scapularis* (420 female, 420 male) and 840 *A. americanum* (420 female, 420 male) ticks. Of these 1680 ticks, 1226 were recovered during the post-attachment period (73%).

**Table 2:** The total number of ticks recovered and their attachment status, feeding status and condition

*Ixodes scapularis*												
Test group	Outcome for introduced ticks by end of 8-day observation period in terms of attached/ detached	Females (*n*)						Males (*n*)		Initial no. ticks placed on deer	No. ticks recovered^a^	Recovery (%)
		Flat		Partially engorged		Fully engorged						
		Alive	Dead	Alive	Dead	Alive	Dead	Alive	Dead			
T48 (48-h FDF exposure)	Attached	0	57	1	45	1	0	1	11	280	210	75
	Detached	0	8	0	3	13	1	0	69			
T120 (120-h FDF exposure)	Attached	0	55	1	19	0	0	0	12	280	221	78.9
	Detached	0	48	0	1	1	0	0	84			
Control	Attached	0	19	7	40	0	0	0	5	280	141	50.4
	Detached	0	2	1	1	45	2	1	18			
*Amblyomma americanum*												
Test group	Outcome for introduced ticks by end of 8-day observation period in terms of attached/ detached	Females (*n*)						Males (*n*)		Initial no. ticks placed on deer	No. ticks recovered^a^	Recovery (%)
		Flat		Partially engorged		Fully engorged						
		Alive	Dead	Alive	Dead	Alive	Dead	Alive	Dead			
T48 (48-h FDF exposure)	Attached	3	31	13	54	0	0	17	41	280	219	78.2
	Detached	0	19	0	3	0	0	26	12			
T120 (120-h FDF exposure)	Attached	1	44	4	50	0	0	3	66	280	199	71.1
	Detached	0	18	2	0	0	0	11	0			
Control	Attached	22	0	94	0	0	0	101	6	280	236	84.3
	Detached	0	0	0	1	0	0	12	0			

These data are for both *Ixodes scapularis* and *Amblyomma americanum* introduced onto white tailed deer fed FDF versus deer fed untreated placebo feed

^a^Recovered ticks refer to ticks collected within the capsules. An additional 49 *I. scapularis* (12 female, 37 male) and 29 *A. americanum* (22 female, 7 male) were found trapped in the veterinary wrap collars. These data were excluded because we could not determine which specific animals these ticks were parasitizing

The probability of *I. scapularis* females detaching or remaining attached was significantly different relative to *A. americanum* (*χ*^2^ = 42.243, *P* < 0.0001), with *I. scapularis* being more likely to detach over the 8-day observation period. Males of both tick species showed a significantly greater tendency to be detached than did females (*χ*^2^ = 273.195, *P* < 0.0001). The probability of ticks being dead was significantly greater for *I. scapularis*, relative to *A. americanum*, over the 8-day observation period (*χ*^2^ = 138.370, *P* < 0.0001).

A total of 572 out of 840 *I. scapularis* were recovered (68.1%) of which 371 (64.9%) were female. Of the recovered females, 66% (*n* = 245) were attached; in contrast, 85.6% of males (*n* = 172) were detached. Recovery within test groups totaled 75% (T48), 78.9% (T120) and 50.4% (control group). Recovery was more difficult within the control group, with a discrepancy in the number of males (*n* = 24), relative to the treatment groups (*n* = 81, *n* = 96). A total of 654 of 840 *A. americanum* ticks were recovered (77.9%), with test groups totaling 78.2% (T48), 71.1% (T120) and 84.3% (control group). The *A. americanum* ticks had a greater tendency to remain attached, with 88% of females (*n* = 316) and 79.3% of males (*n* = 234) still attached by the end of the 8-day post-attachment period.

#### Tick feeding status and condition

Tick feeding status and condition data are explicitly presented in Table 2 and were used to calculate all sums and percentages presented in this section. Within the T48, T120 and control groups, 11.6% (*n* = 15), 1.6% (*n* = 2) and 45.3% (*n* = 53), respectively, of recovered female *I. scapularis* were alive. All flat *I. scapularis* females collected at day 8 post-attachment were dead regardless of test group, with a far greater number of flat females collected in the T48 (*n* = 65) and T120 (*n* = 103) groups than in the control group (*n* = 21). Survivorship was significantly greater within the control group, relative to the treatment groups (*χ*^2^ = 82.696, *P* < 0.0001). Within the T48 and T120 groups, the greatest proportion of *I. scapularis* females collected were flat and dead, representing 50.4% (*n* = 65) and 82.4% (*n* = 103) of the respective totals collected. There was a significant difference in the feeding status of the female ticks of the control group relative to those of T48 and T120 groups (*χ*^2^ = 114.495, *P* < 0.0001). Live and fully engorged females accounted for the largest proportion of ticks within the control group (38.5%, *n* = 45), a by-product of no exposure to FDF, with dead and partially engorged females accounting for the second largest proportion (35%, *n* = 41). Only two of 201 males collected by day 8 post-attachment were alive (1%). For *A. americanum*, in the T48, T120 and control groups, 13% (*n* = 16), 5.9% (*n* = 7) and 99.1% (*n* = 116), respectively, of recovered females were alive. Tick survivorship was significantly greater within the control group relative to the treatment groups (*χ*^2^ = 265.729, *P* < 0.0001). Within the treatment groups, the proportions of flat and dead and partially engorged and dead females were relatively similar and represented the majority of females collected in the T48 (87%, *n* = 107) and T120 (94.1%, *n* = 112) groups. There was a significant difference in the feeding status of female ticks in the control group relative to female ticks in the T48 and T120 groups (*χ*^2^ = 24.967, *p* < 0.0001). Within the control group, 80.3% of the females collected were partially engorged and alive. In total, 57.6% of *A. americanum* males collected were alive at day 8 post-attachment, with 44.8% (*n* = 43), 17.5% (*n* = 14) and 95% (*n* = 113) of males found alive in the T48, T120 and control groups, respectively.

#### *Ixodes scapularis* oviposition and larval hatching

A summary of the average weights of engorged *I. scapularis* females and egg masses, the approximate number of eggs and the approximate number of emerging larvae is presented in Additional file 8: Table S4. Although the control group yielded slightly heavier engorged females and egg masses, and larger numbers of eggs and larvae, relative to the treatment groups, it was determined that these differences were not statistically significant.

### Mortality/efficacy estimates

**Fig. 3:**
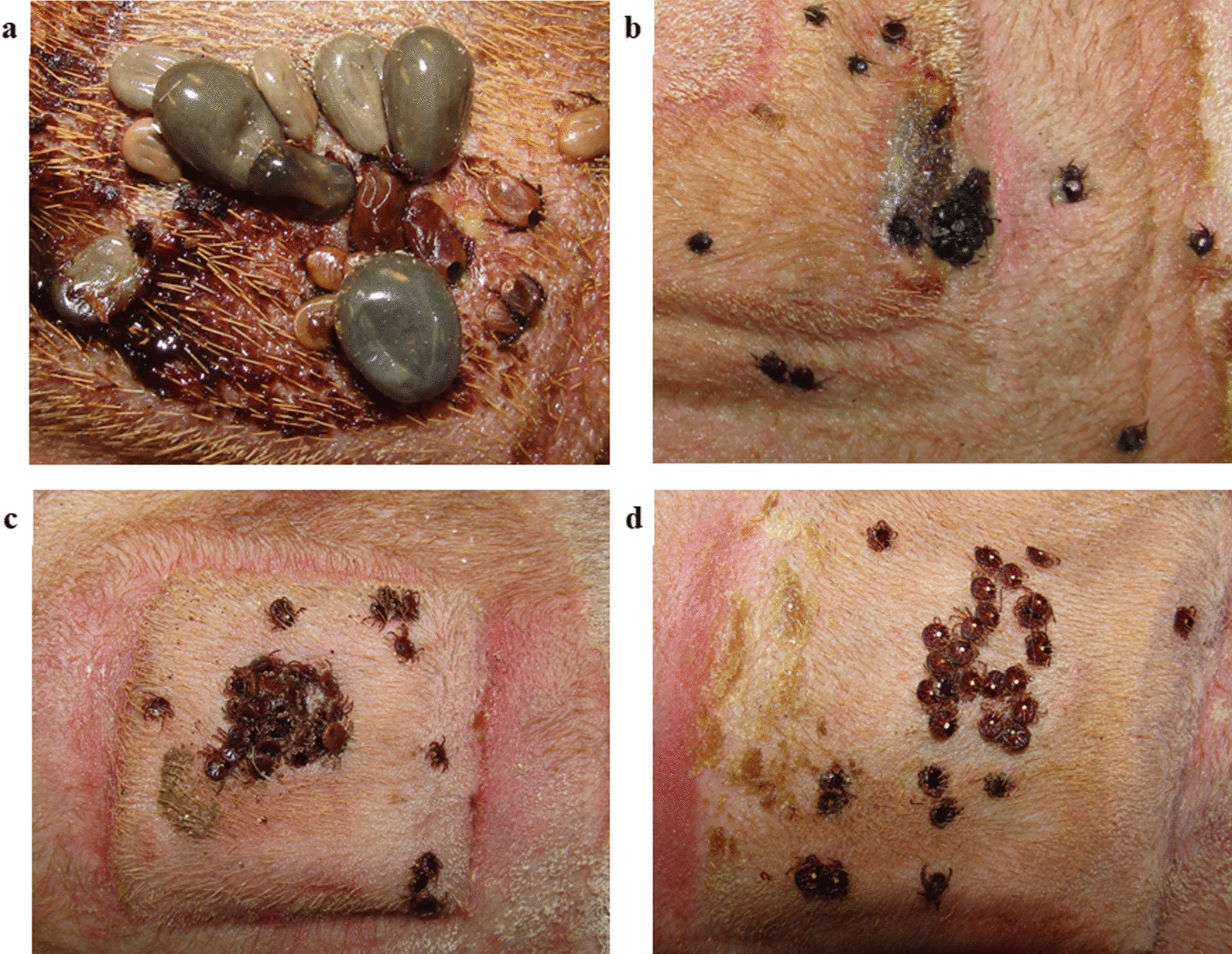
Attached ticks within the capsules of treated and untreated white-tailed deer.** a**,** b** *Ixodes scapularis*: **a** actively feeding on a control deer,** b** dead and attached on a treatment deer.** c**,** d** *Amblyomma americanum***: c** actively feeding on a control deer,** b** dead and attached on a treatment deer. The engorgement rate of *I. scapularis* was faster relative to *A. americanum*. All photos were taken at day 6 post-attachment

Treating deer with FDF had a significant impact on the mortality/survivorship of both tick species. The average number of engorged *I. scapularis* females detaching per deer within each test group and subgroup, and resulting efficacy estimates, are presented in Table 3. *Amblyomma americanum* blood fed markedly slower than *I. scapularis*, and thus efficacy in preventing fully engorged females from detaching was not attainable during the 8 day post-attachment period (Additional file 9: Figure S4). The average number of live female *I. scapularis* and *A. americanum* (attached and detached) observed per deer per test subgroup and resulting efficacy estimates are presented in Table 4. Representative photos of attached *I. scapularis* and *A. americanum* within the control and treatment groups are presented in Fig. 3.

**Table 3:** Fipronil deer feed efficacy in preventing *Ixodes scapularis* females from reaching engorgement and detaching

Test group	FDF exposure time (h)	Number of days post-exposure for tick challenge	Engorged females/deer by day 8 after ticks introduced (average ± SD)	Treatment efficacy (%)
T48	48	7	0.25 ± 0.50	95.6
		21	3.00 ± 3.16	46.7
		Cumulative	1.63 ± 2.56	71.1
T120	120	7	0	100
		21	0.25 ± 0.50	95.6
		Cumulative	0.13 ± 0.35	97.8
Control			5.63 ± 4.98	

*SD *Standard deviation

**Table 4:** Fipronil deer feed efficacy in reducing *Ixodes scapularis* and *Amblyomma americanum* survivorship

Test group	FDF exposure time (h)	Days post-exposure for tick challenge	Mean live females/deer by day 8 after ticks introduced^a^	Treatment efficacy (%)
*Ixodes scapularis*				
T48	48	Day 7	0.25 ± 0.50	96.2
		Day 21	3.50 ± 3.70	47.2
T120	120	Day 7	0.00 ± 0.00	100
		Day 21	0.50 ± 1.00	92.5
Control			6.63 ± 5.78	
*Ambloymma americanum*				
T48	48	Day 7	1.50 ± 2.38	89.7
		Day 21	2.50 ± 3.70	82.8
T120	120	Day 7	0.00 ± 0.00	100
		Day 21	1.75 ± 2.87	87.9
Control			14.50 ± 4.66	

^a^Includes attached and detached females alive at day 8 post-attachment. Values are the average ± SD

### FDF efficacy against *Ixodes scapularis*

**Fig. 4:**
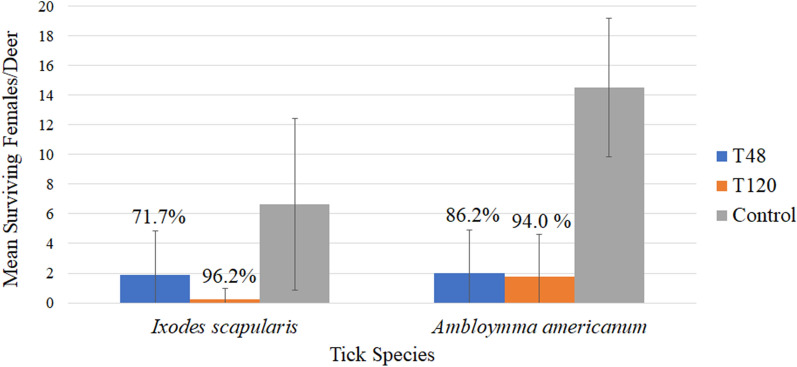
Fipronil deer feed (FDF) efficacy in reducing *Ixodes scapularis* and *Amblyomma americanum* survivorship in the treatment groups. T48, Treatment group exposed to FDF for 48 h (2 days) ; T120, treatment group exposed to FDF for 120 h (5 days)

Treating deer with the FDF for 48 h resulted in 95.6% efficacy in preventing ticks placed on deer on day 7 post-FDF exposure from feeding to engorgement and detaching, relative to the control group (Table 3). It also led to a 96.2% reduction in survivorship of females, relative to the control group (Table 4). Efficacy of 48-h FDF treatment dropped for ticks placed on deer on day 21 post-FDF exposure, with a 46.7% reduction in detaching engorged ticks (Table 3) and a 47.2% reduction in survivorship of females (Table 4), relative to the control group. Treating deer with FDF for 120 h resulted in 100% efficacy in reducing female survivorship (Table 4) and in preventing ticks placed on deer on day 7 post-FDF exposure from feeding to engorgement and detaching (Table 3). For ticks placed on deer on day 21 post-FDF exposure, the 120-h FDF treatment efficacy in reducing detached engorged ticks and overall survivorship was 95.6% and 92.5%, respectively. When combining the subgroups for timing of tick placement on deer, the 48-h FDF treatment resulted in a 71.1% reduction in detached engorged females (Table 3) and a 71.7% reduction in survivorship (Fig. 4), and the 120-h FDF treatment resulted in a 97.8% reduction in engorgement and detachment (Table 3) and a 96.2% reduction in survivorship (Fig. 4).

### FDF efficacy against *Amblyomma americanum*

Treating deer with the FDF for 48 h resulted in an efficacy of 89.7% and 82.8% when ticks were placed on deer on day 7 and day 21 post-FDF exposure, respectively (Table 4). Treating deer with FDF for 120 h resulted in an efficacy of 100% and 87.9% when ticks were placed on deer on day 7 and day 21 post-FDF exposure, respectively (Table 4). When combining subgroups for timing of tick placement on deer, the 48-h and 120-h FDF treatments resulted in 86.2% and 94% reductions in survivorship, respectively (Fig. 4).

### Fipronil concentrations in deer

The majority of pure fipronil was metabolized or excreted. Fipronil sulfone was the metabolite detected above the LOQ.

The *Cp* values for each test deer are presented in Table 5. All treated deer had fipronil sulfone detectable > LOQ. The *Cp* values were highest in the 120-h exposure group (T120), with the average *Cp* being 57.3 ppb (day 7 post-FDF exposure) and 21.7 ppb (day 21 post-FDF exposure). For the 48-h exposure group (T48), the average *Cp* values were 20.1 ppb (day 7 post-FDF exposure) and 7.6 ppb (day 21 post-FDF exposure). The reduction from day 7 to day 21 after FDF exposure supports the reduction in efficacy observed. There was a significant linear correlation between *Cp* and the mg/kg fipronil that was consumed by individual deer (*r*^2^ = 0.6150; *P* < 0.0001). Additionally, there were correlations between *Cp* and the number of surviving female *I. scapularis* (*r*^2^ = 0.2057; *P* < 0.0260) and *A. americanum* (*r*^2^ = 0.3573; *P* < 0.0020) per deer. However, tick survivorship appeared to decrease exponentially rather than linearly in response to elevated *Cp*, with no female ticks surviving when *Cp* in plasma was ≥ 25.0 ppb.

**Table 5:** Fipronil sulfone concentrations in plasma (*Cp*) for individual white-tailed deer

Test group	Sex	Body weight (kg)	Amount fipronil/body weight (mg/kg)	Days post-FDF exposure	Fipronil (ppb)	Fipronil sulfone (ppb)	Number of female ticks alive at day 8 after tick introduction
T48 (48-h FDF exposure)	Female	83.8	0.6	7	< LOQ	16.8	1
	Male	86.4	0.58	7	0.7	32.2	0
	Female	72.2	0.32	7	0.5	6.3	6
	Male	93.9	0.27	7	0.2	25.0	0
	Male	77.9	0.27	21	< LOQ	2.5	8
	Female	82.4	0.24	21	< LOQ	2.4	8
	Male	50.4	0.99	21	< LOQ	12.2	6
	Female	72.1	0.34	21	< LOQ	13.2	2
T120 (120-h FDF exposure)	Male	104	1.2	7	2.5	74.3	0
	Female	81.8	1.24	7	0.9	89.7	0
	Female	56.8	1.1	7	< LOQ	37.7	0
	Female	68.4	0.91	7	1.5	27.3	0
	Female	66.3	0.93	21	< LOQ	26.4	0
	Female	62.7	0.94	21	< LOQ	5.6	8
	Female	50.9	1.47	21	< LOQ	41.1	0
	Female	84.1	0.44	21	< LOQ	13.5	1
Control	Male	99.9	NA	7	ND	ND	32
	Female	78.8	NA	7	ND	ND	14
	Male	74.2	NA	7	ND	ND	26
	Female	52.8	NA	7	ND	ND	34
	Female	69.3	NA	21	ND	ND	10
	Male	83.5	NA	21	ND	ND	11
	Female	91.3	NA	21	ND	ND	17
	Male	79.2	NA	21	ND	ND	25

These data represent all deer fed FDF and untreated control deer (fed placebo), and the subsequent number of live female ticks (attached and detached *I. scapularis* and *A. americanum*) at the conclusion of the tick challenge on day 8 after tick introduction (*n* = 40 females per deer; 20 females for each species)

*LOQ* Limit of detection, *ND* no fipronil detected

Explicit *Cf* values for each deer are available in Additional file 10: Table S5. All treated deer had *Cf* > LOQ. The *Cf* values were highest in the 120-h exposure group (T120), with the average *Cf* being 108.8 ppb (day 7 post-FDF exposure) and 48.7 ppb (day 21 post-FDF exposure). For the 48-h exposure (T48), the average *Cf* values were 44.8 ppb (day 7 post-FDF exposure) and 28.1 ppb (day 21 post-FDF exposure). Similar to *Cp*, there was a significant linear correlation between *Cf* and the mg/kg fipronil that was consumed by individual deer (*r*^2^ = 0.5680; *P* < 0.0001).

**Fig. 5:**
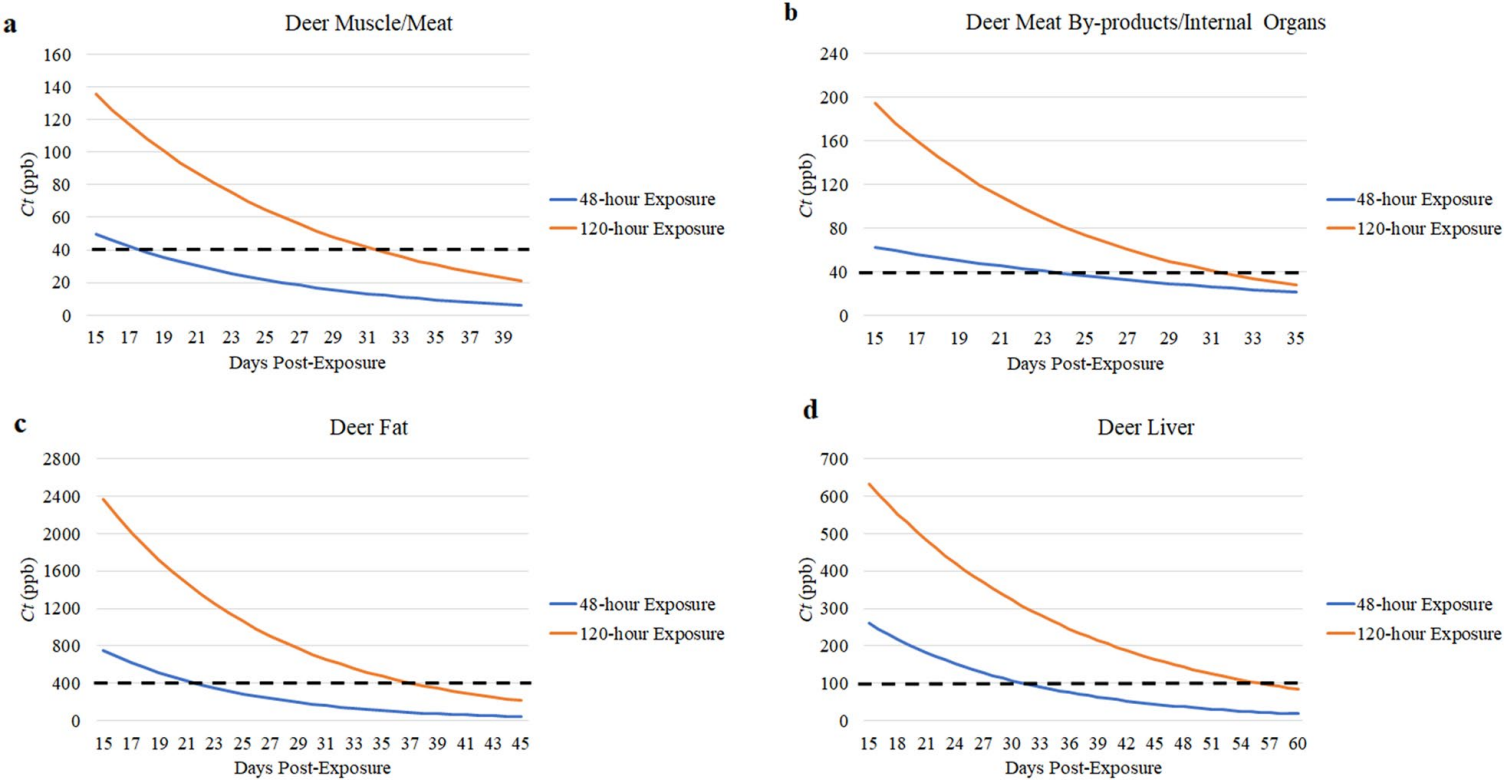
The predicted degradation of fipronil sulfone in various deer tissue classifications. Fipronil degradation in meat/muscle (**a**), meat by-products (**b**), fat (**c**) and liver (**d**) of white-tailed deer after consumption of fipronil deer feed. The dotted line indicates the maximum residue limits established for each tissue classification

The *Ct* values were significantly different among various tissue classifications (*χ*^2^ = 81.591, *df* = 3, *P* < 0.0001). Fipronil is a lipophilic compound [48], and the *Ct* in fat was determined to be significantly higher relative to that in meat/muscle tissues (*Z* =  − 7.905, *P* < 0.0001), meat by-products (*Z* =  − 5.906, *P* < 0.0001) and liver (*Z* =  − 2.516*, P* = 0.0119). Additionally, *Ct* values in liver tissues were significantly greater than the *Ct* values in meat (*Z* =  − 4.918, *P* < 0.0001) and meat by-products (*Z* =  − 3.816, *P* < 0.0006). Deer exposed to fipronil for 120 h had significantly higher *Ct* in fat (*Z* = 2.848, *P* = 0.0044), meat (*Z* = 5.521, *P* < 0.0001) and meat by-products (*Z* = 2.224, *P* = 0.0261) (liver was not significant), relative to deer exposed to fipronil for 48 h. A summary of the *Ct* in tissues is given in Additional file 11: Table S6. *Ct* was present > LOQ in all tissues collected from deer in the treatment groups. For T48 (48-h exposure)*,* differences in *Ct* values obtained from tissues collected at day 15 and day 29 were significant (*Z* = − 4.873, *P* < 0.0001), with *Ct* values at day 29 being 74% (fat), 56% (liver), 68.9% (meat) and 52.8% (meat by-products) less than at day 15. The difference in *Ct* between day 15 and day 29 was significant (*Z* = 4.287, *P* < 0.0001) in the T120 (120-h exposure) group also, with *Ct* at day 29 being 67.5% (fat), 46.7% (liver), 64.7% (meat) and 74.2% (meat by-products) less than at day 15. Our estimates suggested that the respective deer exposed to FDF for 48 h and 120 h would have post-exposure *Ct* values degrade to below the EPA MRLs within 22 and 38 days (fat), 32 and 56 days (liver), 18 and 32 days (meat) and 24 and 32 days (meat by-products), respectively (Fig. 5).

## Discussion

White-tailed deer represent a key reproductive host for *I. scapularis* and *A. americanum*, and host-targeted approaches such as the application of an oral acaricide have the potential to dramatically reduce tick population abundance and subsequent risk of pathogen-infected tick bites. The results of this study suggest that a deer feed containing a nominal concentration of 0.0025% fipronil, presented to white-tailed deer for 48 h and 120 h, can control female *I. scapularis* and *A. americanum* ticks parasitizing at day 7 and day 21 post-exposure, with the efficacy dependent upon the amount consumed and the subsequent concentration of fipronil sulfone in the plasma. The fact that we detected no significant differences when comparing FDF and placebo consumption suggested that the inclusion of fipronil in the formulation did not reduce feed palatability. Our results suggest that 100% of *I. scapularis* and *A. americanum* were eliminated when *Cp* was present at ≥ 25 ppb. Additionally, in total, only one live female tick (*A. americanum*) was collected from a deer having *Cp* at 13.5 ppb. The current *Cp* values are higher than the *Cp* values determined to control 100% *I. scapularis* larvae parasitizing *P*. *leucopus* (≥ 8.8 ppb) [27] but are markedly lower than the fluralaner concentrations in plasma reportedly required to control 97% (13000 ppb) and 94% (4000 ppb) *I. scapularis* larvae parasitizing *Peromyscus maniculatus* (a species closely phylogenetically related to *P*. *leucopus*) [50]. The integration of rodent-targeted and deer-targeted oral acaricide usage would have the potential to reduce tick burden on the primary pathogen host and key reproductive hosts for the *I. scapularis* vector of *B. burgdorferi* and, if properly implemented, could potentially have a significant impact on vector abundance and *B. burgdorferi* infection prevalence in the areas where applied.

The efficacy of FDF in controlling ticks at day 7 post-exposure was undeniable, with 48-h and 120-h exposure successfully controlling both tick species. For *I. scapularis*, near-complete control was obtained, with only one detached engorged *I. scapularis* being collected in total (48-h exposure only). The treatment was also effective against *A. americanum*, with complete control obtained in the 120-h exposure group and nearly 90% control within the 48-h exposure group. At day 21 post-exposure, 120-h fipronil exposure resulted in a high efficacy against *I. scapularis,* with only one detached engorged female being collected in total. The effectiveness of the 48-h exposure and 120-h exposure against *A. americanum* were relatively similar at day 21 with > 80% efficacy for each. *Amblyomma americanum* blood feed for an extended duration, relative to *I. scapularis*, with approximately 70% of females of the former species needing 12–16 days to reach full engorgement [51], which is why the *A. americanum* we utilized had a greater tendency, relative to *I. scapularis*, to remain attached throughout the 8-day post-attachment period. Thus, we were not able to monitor this species until full engorgement and detachment. Considering these females may have fed for an additional 4–8 days, we strongly suspect that efficacy would have increased and may have reached 100% within all treatment groups. For the purposes of this proof-of-concept laboratory experiment, it was determined that both tick species could be evaluated concurrently. However, if explicit engorgement and detachment data are desired for *A. americanum* in the future, researchers will need to consider exclusively evaluating this species and extending the post-attachment period by several days. The above values satisfy efficacy requirements previously outlined by the EPA for federal approval which suggest an efficacy of 80–100% against tick vectors [39]. If the product proves to be palatable under field conditions as well, and thus can reach a sizable proportion of deer, the fact that FDF was effective up to day 21 post-exposure indicates that the product could be utilized relatively infrequently under field conditions, which would reduce the amount of acaricide going into the environment, thereby reducing risk of exposure to non-target species and bioaccumulation. From a management perspective, the above results are encouraging.

Direct resistance of host-seeking arthropods to fipronil is considered improbable [49, 52], and fipronil’s effectiveness at low concentrations allows for reduced application rates which pose reduced risk to non-target organisms, relative to other candidate compounds such as malathion and carbaryl [53]. The acute oral LD_50_ (lethal dose for 50% of test animals/subjects) of fipronil reported for representative mammal species is 97 mg/kg (rats) [54]. Fipronil consumption by deer in the current study ranged from 0.24 mg/kg to 0.99 mg/kg for the 48-h exposure and 0.44 mg/kg to 1.47 mg/kg for the 120-h exposure (Table 1). The rate of fipronil exposure is reduced because of the low concentration of fipronil in FDF (0.0025%). As an example, a deer in the T120 group weighing 104 kg consumed 5 kg of FDF, which amounted to 1.2 mg/kg fipronil consumed (Table 1). This same deer would need to consume > 403 kg of FDF in one sitting to ingest enough fipronil to exceed the oral LD_50_ for mammalian species, a feat that would be highly improbable. The ability of FDF to be applied at relatively low frequencies, in combination with the low dose of fipronil in the formulation, reduces the risk to non-target species, such as raccoons, rodents or birds, should they come into contact with FDF. However, field applications will be conducted using elevated, species-specific deer feeders to considerably reduce or prohibit access by non-target species, including potentially more sensitive animal species, such as rabbits [55]. Future studies involving field deployment of FDF should carefully consider the application rates and explicitly monitor non-target species within treated areas to ensure reduced environmental risk. Considering that fipronil has proven to be effective against vectors of human disease, such as fleas, mosquitoes and phlebotomine sand flies, a focus of future research may include investigating the impact of fipronil treatment on other blood-feeding arthropods associated with deer, such as mosquito species [56] Ceratopogonidae [57] and deer keds [58].

While the efficacy results of our experiment meet federal recommendations, LC/MS analysis of tissues suggest that the concentrations of fipronil sulfone in various tissues should be further investigated and that appropriate modifications to management plans should be considered. Our results suggest that fipronil sulfone levels will fall below the EPA-established MRL values within a moderate time frame, with fipronil degrading most slowly in the liver. To our knowledge, the US FDA and US EPA do not have established MRL values for deer. Thus, we considered MRL values for ruminant cattle to be the most appropriate when evaluating the results. Venison liver consumption in general is not recommended due to possible liver fluke (*Fascioloides magna*) infection [59, 60] or contamination from heavy metals, such as cadmium [61, 62]. We note that the deer in this study were raised in captivity. Factors such as environmental conditions, forage availability and stressors may influence fat stores, digestion and metabolic rates in white-tailed deer [63, 64], and these factors may differ in wild populations, relative to those raised in captivity. Thus, fipronil metabolism and degradation in wild deer may differ from that in captive deer, and this may be worth investigating in future research. By using these collected data to attempt to predict exponential degradation of fipronil, we can make some initial assumptions to aid in management plans. If the treatment strategies in this pen study are utilized in the field, FDF may work most efficiently in states where Lyme disease is endemic and where there are sizable peaks of adult *I. scapularis* in the spring [65], with simultaneous targeting of peaks in *A. americanum*. Treating in the spring and early to mid-summer would allow adequate time for fipronil withdrawal in deer tissues prior to the fall deer hunting season, which coincides with a peak in fall activity of *I. scapularis* adults. Two factors make *A. americanum* an ideal target for FDF treatment: (i) all three life stages of *A. americanum* take blood meals from white-tailed deer [6]; and (ii) each life stage has peak activity which occurs outside of the fall deer hunting season [66–68]. Additionally, modifications may be made in specific scenarios, such as blending FDF with untreated placebo or whole corn to dilute the fipronil concentration. A lower fipronil concentration (such as 0.0005%) could allow for FDF to be presented for extended durations under field conditions concurrently with peak adult tick activity or could potentially increase the potential for reduced/short-term treatment during hunting season. Agent-based vector-host associative modeling [69] could be useful in predicting the potential of various FDF treatment scenarios. Ultimately, a field trial will be necessary to confirm the concentrations of fipronil sulfone in the tissues of wild deer and to determine the best course of action for future management plans that might involve the use of FDF.

We intend to investigate possible alternative treatment approaches in regions where concerns regarding chronic wasting disease (CWD) and bovine tuberculosis (bTB) preclude deer baiting. The concern is centered primarily upon unnatural congregation of large groups of deer that can result from baiting, which can subsequently increase the risk of direct contact transmission of CWD and bTB [70, 71]. Similar to vaccine deployment strategies being explored in the Midwest USA [72], an alternative approach being considered is to develop a large grid of evenly placed feeders, each containing a small quantity of FDF (approx. 0.5 kg) to target a few deer, rather than large groups, thus reducing congregation. The logistics of such a strategy will need to be further explored during future field testing.

In past studies, many researchers have explicitly described difficulties related to manual tick feeding on animals [26, 73, 74]. The capsule sizes and dimensions we utilized were adequate to maintain 20 mating pairs of *I. scapularis*, as the surface area of our capsules (7 × 7 cm) and the capsule depth (18 mm) exceeded the surface area (5 × 5 cm) and capsule depth (8 mm) of similar capsules used for infesting rabbits with 20 *I. scapularis* mating pairs [44]. While we were relatively successful in recovering ticks on the deer, we still had issues with damage or loss of *I. scapularis*, particularly within the control group. Because FDF increased mortality within the treatment groups, deer in the treatment groups presumably experienced reduced irritation with fewer actively engorging females, which made the deer less likely to attempt to dislodge the capsules. Given the small size of *I. scapularis* males, minor shifting of the capsules within the control group apparently was enough to promote escape. The increased irritation caused by actively engorging females, and subsequent shifting of capsules, caused many females to become damaged/popped, or asphyxiated in the coagulated blood of popped females, which resulted in an increased number of dead, partially engorged females. Thus, the formula described by Abbott [47] was critical in estimating the efficacy by accounting for tick survivorship in the control group. This was not an issue with *A. americanum*, given they feed much more slowly. However, while our capsule depth exceeded the specifications recommended by Almazan et al. [44] (10 mm), we suspect *A. americanum* damage may have become a considerable issue if they fed to engorgement considering they are a much larger tick, weighing > 600 mg on average at full engorgement [75, 76]. Researchers may want to make additional modifications to these capsules in future studies to continue to improve tick attachment, recovery and survivorship. While the issues described limited our ability to determine significant FDF impact on oviposition success and larval emergence, it is worth reiterating that the approximate eggs laid/female and the approximate larvae/female within the treatment groups were slightly reduced, relative to those of the control group, and that this may want to be investigated further in future studies. If a field trial is performed in which FDF is presented to wild deer, researchers might consider removing engorged females from treated deer in the wild and monitoring them for oviposition success.

## Conclusions

The results of the present study demonstrate the potential usefulness of a fipronil-based oral acaricide in controlling at least two medically important tick species by providing insights into the potential for control up to 21 days post-exposure. The results on fipronil residue in tissues, plasma and feces also provide much needed insights into the fate of fipronil and its metabolites and provide a potential blueprint for future trials. The next logical step would be a field trial in which wild deer would be exposed to FDF at pre-determined exposure points. Deer would then be sedated in the field and ectoparasites would be collected to determine the impact on parasitizing tick burden. A subsample of deer would also be euthanized, and tissues would be obtained to determine the fate of fipronil in wild deer. If this proof-of-concept field work proves successful, a multi-year large-scale field trial would be the next logical step. Confirming efficacy and safety of this product under field conditions could pave the way for federal approval of a product available for large-scale tick control. A fipronil product targeting wild ruminants on a broad scale has the potential for a variety of beneficial applications. Previous research has indicated the effective use of low-dose oral fipronil baits in controlling ticks [26, 27], fleas [23–25], sand flies [28, 29] and mosquitoes [22, 30] parasitizing a variety of mammalian hosts, including rodents and ruminant cattle. Other tick species of medical or veterinary importance, such as cattle ticks (*Rhipicephalus* *annulatus*, *R. microplus*) and *H. longicornis*, also utilize white-tailed deer as a blood-meal host [7, 8] and would be susceptible to this treatment as well. Thus, researchers should explore usage of FDF for controlling other arthropod vectors beyond those investigated in the current study. The ultimate goal is to produce a federally approved product for use in controlling arthropods infesting white-tailed deer. If US FDA approval is granted for a new animal drug, FDF could provide a useful means of controlling multiple arthropod species, parasitizing wild ruminants, capable of vectoring disease agents transmissible to humans.

## Supplementary Information

**Additional file 1. Table S1. **Group specifics. Summary of the test group deer utilized during the pen study and fed fipronil deer feed (FDF) or a placebo deer feed.

**Additional file 2. Figure S1. **Fipronil deer feed presentation. FDF presented in an elevated deer feeder during the exposure period.

**Additional file 3. Figure S2. **Tick capsule.

**Additional file 4. Video S1. **Tick application. *Amblyomma americanum* (20 mating pairs) being inserted into a capsule attached to a test deer.

**Additional file 5. Figure S3. **Deer in individual pens with completed, attached capsules.

**Additional file 6. Table S2. **Tissue details. Classification, US Environmental Protection Agency-established maximum residue limits (MRL) and tissue identification for all tissues collected from all euthanized deer.

**Additional file 7. Table S3. **Study schedule. Specific timing of acclimation, exposure, tick attachment, post-attachment, capsule checks and tissue collection are presented for each deer.

**Additional file 8. Table S4. ***Ixodes scapularis* eggs and larvae. The average ± standard deviation (SD) weights for engorged *I. scapularis* females and approximate number of eggs and larvae produced within FDF treatment and control groups.

**Additional file 9. Figure S4. ***Ixodes scapularis* and *Amblyomma americanum* feeding on a control deer. Although *A. americanum* is a larger tick than *I. scapularis*, the engorgement rate is markedly slower.

**Additional file 10. Table S5. ***Cf* values for each white-tailed deer.

**Additional file 11. Table S6. **Fipronil sulfone in white-tailed deer tissues.

## Data Availability

The datasets generated during and/or analyzed during the current study are available from the corresponding author upon reasonable request.
