# Supplementary material for: Development of a low-dose fipronil deer feed: evaluation of efficacy against two medically important tick species parasitizing white-tailed deer (Odocoileus virginianus) under pen conditions
Source: Parasit Vectors. 2023 Mar 9;16:94. doi: 10.1186/s13071-023-05689-1 (PMC9999526; doi:10.1186/s13071-023-05689-1)
Supplement: Supplementary file 1 — Additional file 1. Table S1. Group specifics. Summary of the test group deer utilized during the pen study and fed fipronil deer feed (FDF) or a placebo deer feed. [file 13071_2023_5689_MOESM1_ESM.docx]

**Table 1.** Summary of the test group deer utilized during the pen study and fed fipronil deer feed (FDF) or a placebo deer feed.

| Test Group ID | Feed Presented | Feed Exposure Duration | Tick Attachment Timepoint (Post-feed exposure) | Individual Test Deer ID | Euthanized For Tissue Collection (*y/n*) |
| --- | --- | --- | --- | --- | --- |
| T48 | FDF | 48-hour | Day-7 | T1-1 | *y* |
|  |  |  |  | T1-2 | *y* |
|  |  |  |  | T1-3 | *y* |
|  |  |  |  | T1-4 | *y* |
|  |  |  | Day-21 | T1-5 | *y* |
|  |  |  |  | T1-6 | *y* |
|  |  |  |  | T1-7 | *y* |
|  |  |  |  | T1-8 | *y* |
| T120 | FDF | 120-hour | Day-7 | T2-1 | *y* |
|  |  |  |  | T2-2 | *y* |
|  |  |  |  | T2-3 | *y* |
|  |  |  |  | T2-4 | *y* |
|  |  |  | Day-21 | T2-5 | *y* |
|  |  |  |  | T2-6 | *y* |
|  |  |  |  | T2-7 | *y* |
|  |  |  |  | T2-8 | *y* |
| Control | Placebo Deer Feed | 48-hour | Day-7 | C-1 | *n* |
|  |  |  |  | C-2 | *n* |
|  |  |  |  | C-3 | *y* |
|  |  |  |  | C-4 | *n* |
|  |  | 120-hour | Day-21 | C-5 | *y* |
|  |  |  |  | C-6 | *n* |
|  |  |  |  | C-7 | *n* |
|  |  |  |  | C-8 | *n* |

**y/n*= yes/no
