# Supplementary figures and images for: Development of a low-dose fipronil deer feed: evaluation of efficacy against two medically important tick species parasitizing white-tailed deer (Odocoileus virginianus) under pen conditions
Source: Parasit Vectors. 2023 Mar 9;16:94. doi: 10.1186/s13071-023-05689-1 (PMC9999526; doi:10.1186/s13071-023-05689-1)

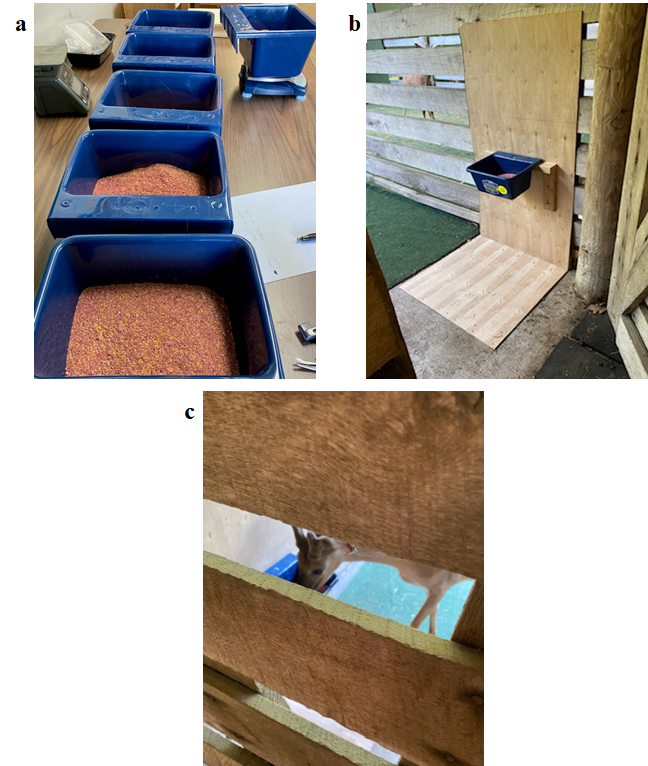

Supplement: Supplementary file 2 — Additional file 2. Figure S1. Fipronil deer feed presentation. FDF presented in an elevated deer feeder during the exposure period. [file 13071_2023_5689_MOESM2_ESM.tiff]

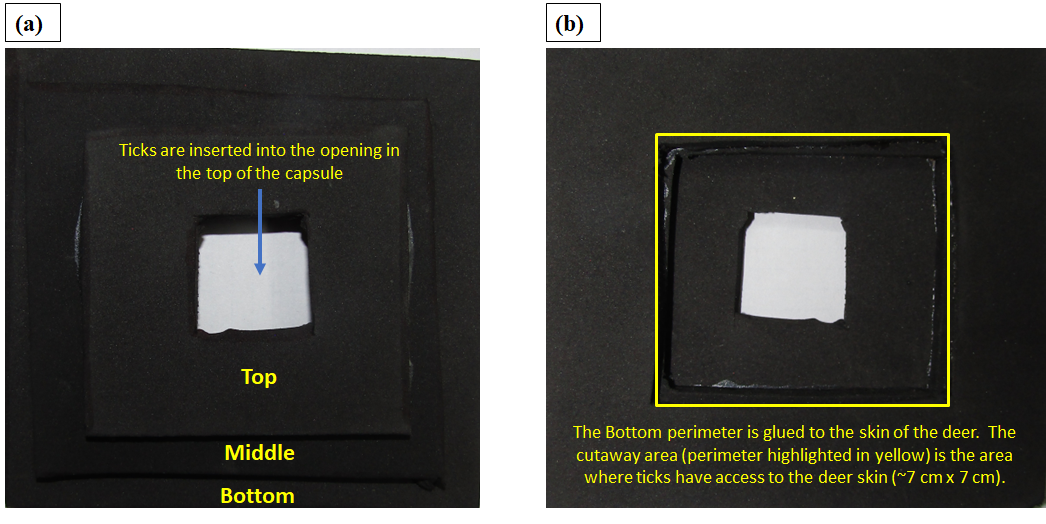

Supplement: Supplementary file 3 — Additional file 3. Figure S2. Tick capsule. [file 13071_2023_5689_MOESM3_ESM.tiff]

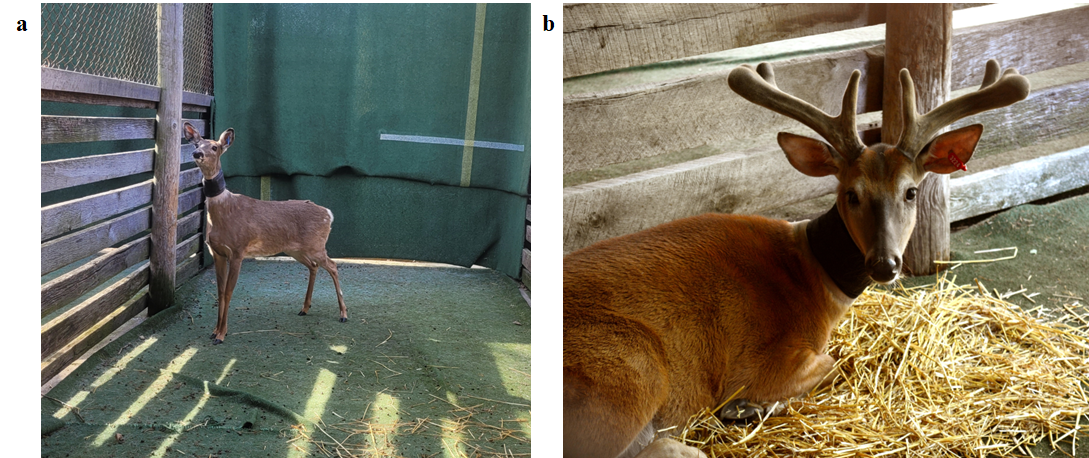

Supplement: Supplementary file 5 — Additional file 5. Figure S3. Deer in individual pens with completed, attached capsules. [file 13071_2023_5689_MOESM5_ESM.tiff]

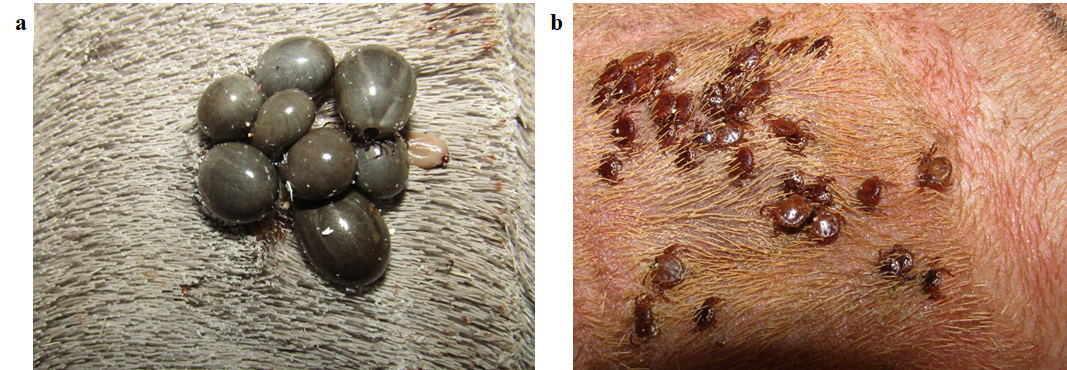

Supplement: Supplementary file 9 — Additional file 9. Figure S4. Ixodes scapularis and Amblyomma americanum feeding on a control deer. Although A. americanum is a larger tick than I. scapularis, the engorgement rate is markedly slower. [file 13071_2023_5689_MOESM9_ESM.tiff]
