# Supplementary material for: Development of a low-dose fipronil deer feed: evaluation of efficacy against two medically important tick species parasitizing white-tailed deer (Odocoileus virginianus) under pen conditions
Source: Parasit Vectors. 2023 Mar 9;16:94. doi: 10.1186/s13071-023-05689-1 (PMC9999526; doi:10.1186/s13071-023-05689-1)
Supplement: Supplementary file 6 — Additional file 6. Table S2. Tissue details. Classification, US Environmental Protection Agency-established maximum residue limits (MRL) and tissue identification for all tissues collected from all euthanized deer. [file 13071_2023_5689_MOESM6_ESM.docx]

Tissue classification, Environmental Protection Agency-established maximum residue limits (MRL), and tissue identification for all tissues collected from all euthanized deer.

| Tissue Classification | *MRL | Tissue Identification |
| --- | --- | --- |
| Fat | 400 ppb | Subcutaneous fat |
|  |  | Abdominal fat |
| Meat/Muscle | 40 ppb | Neck |
|  |  | Backstrap |
|  |  | Rump |
|  |  | Round |
|  |  | Flank |
|  |  | Shank |
|  |  | Shoulder |
|  |  | Rib meat |
| Meat Biproducts | 40 ppb | Heart |
|  |  | Kidney |
|  | 100 ppb | Liver |
