# Supplementary material for: Development of a low-dose fipronil deer feed: evaluation of efficacy against two medically important tick species parasitizing white-tailed deer (Odocoileus virginianus) under pen conditions
Source: Parasit Vectors. 2023 Mar 9;16:94. doi: 10.1186/s13071-023-05689-1 (PMC9999526; doi:10.1186/s13071-023-05689-1)
Supplement: Supplementary file 8 — Additional file 8. Table S4. Ixodes scapularis eggs and larvae. The average ± standard deviation (SD) weights for engorged I. scapularis females and approximate number of eggs and larvae produced within FDF treatment and control groups. [file 13071_2023_5689_MOESM8_ESM.docx]

The average ± SD weights for engorged *Ixodes scapularis* females and approximate number of eggs and larvae produced within fipronil deer feed (FDF) Treatment and Control groups.

| Test Deer | Engorged Female Weight (g) | Egg Mass Weight (g) | No. Eggs | Proportion Eggs Hatched | No. Larvae |
| --- | --- | --- | --- | --- | --- |
| Placebo Feed Control | 0.1790 | 0.0839 | 1678.5 | 0.92 | 1603.2 |
|  | ±0.0685 | ±0.0406 | ±811.2 | ±0.11 | ±845.1 |
| *FDF Treatment | 0.1765 | 0.0775 | 1550.2 | 0.86 | 1346.6 |
|  | ±0.0524 | ±0.0539 | ±1078.5 | ±0.12 | ±980.5 |

*Only one *I. scapularis* female fed to full engorgement and detached within the 120-hour FDF-exposure group. Thus, no division was made between Treatment groups when conducting this analysis.
