# Supplementary material for: Development of a low-dose fipronil deer feed: evaluation of efficacy against two medically important tick species parasitizing white-tailed deer (Odocoileus virginianus) under pen conditions
Source: Parasit Vectors. 2023 Mar 9;16:94. doi: 10.1186/s13071-023-05689-1 (PMC9999526; doi:10.1186/s13071-023-05689-1)
Supplement: Supplementary file 11 — Additional file 11. Table S6. Fipronil sulfone in white-tailed deer tissues. [file 13071_2023_5689_MOESM11_ESM.docx]

The fipronil sulfone concentrations in various white-tailed deer tissues after consumption of fipronil deer feed (FDF).

| Group | Tissue Classification | Day Post-FDF Exposure | Mean (ppb) ± SD |
| --- | --- | --- | --- |
| T48 (48-hour FDF exposure) | Fat | 15 | 750.0 ± 363.1 |
|  |  | 29 | 194.6 ± 215.1 |
|  | Liver | 15 | 261.0 ± 118.9 |
|  |  | 29 | 114.8 ± 114.4 |
|  | Meat | 15 | 49.4 ± 30.8 |
|  |  | 29 | 15.4 ± 15.1 |
|  | Meat Biproducts | 15 | 63.2 ± 29.9 |
|  |  | 29 | 29.8 ± 42.6 |
| T120 (120-hour FDF exposure) | Fat | 15 | 2350.9 ± 1154.2 |
|  |  | 29 | 762.8 ± 443.2 |
|  | Liver | 15 | 632.6 ± 223.0 |
|  |  | 29 | 336.9 ± 198.7 |
|  | Meat | 15 | 134.9 ± 79.5 |
|  |  | 29 | 47.7 ± 26.2 |
|  | Meat Biproducts | 15 | 194.8 ± 120.1 |
|  |  | 29 | 50.2 ± 26.0 |
